# Anesthesia with Propofol versus Sevoflurane: Does the Longer Neuromuscular Block under Sevoflurane Anesthesia Reduce Laryngeal Injuries?

**DOI:** 10.1155/2013/723168

**Published:** 2013-02-27

**Authors:** Thomas Mencke, Amelie Zitzmann, Susann Machmueller, Arne Boettcher, Martin Sauer, Hans-Wilhelm Pau, Gabriele Noeldge-Schomburg, Steffen Dommerich

**Affiliations:** ^1^Department of Anesthesia and Intensive Care Medicine, University of Rostock, Schillingallee 35, 18057 Rostock, Germany; ^2^Department of Otorhinolaryngology, University of Rostock, 18057 Rostock, Germany

## Abstract

Anesthesia can be maintained with propofol or sevoflurane. Volatile anesthetics increase neuromuscular block of muscle relaxants. We tested the hypothesis, that sevoflurane would cause less vocal cord injuries than an intravenous anesthesia with propofol. In this prospective trial, 65 patients were randomized in 2 groups: SEVO group, anesthesia with sevoflurane, and TIVA group, total intravenous anesthesia with propofol. Intubating and extubating conditions were evaluated. Vocal cord injuries were examined by stroboscopy before and 24 and 72 h after surgery; hoarseness and sore throat were assessed up to 72 h after surgery. Hoarseness and sore throat were comparable between both groups (not significant). Similar findings were observed for vocal cord injuries: 9 (SEVO) versus 5 (TIVA) patients; *P* = 0.36; the overall incidence was 24%. Type of vocal cord injuries: 9 erythema and 5 edema of the vocal folds. Neuromuscular block was significantly longer in the SEVO group compared with the TIVA group: 71 (range: 38–148) min versus 52 (range: 21–74) min; *P* < 0.001. Five patients (TIVA group) versus 11 patients (SEVO group) needed neostigmine to achieve a TOF ratio of 1.0 (*P* = 0.14). Under anesthesia with propofol laryngeal injuries were not increased; the risk for residual curarization, however, was lower compared with sevoflurane.

## 1. Introduction

We showed that tracheal intubation with atracurium significantly decreased vocal cord injuries compared with tracheal intubation without muscle relaxants (8% versus 42%) [1]. Tracheal intubation with atracurium at maximum neuromuscular block, however, did not decrease vocal cord injuries compared with tracheal intubation two minutes after injection of atracurium; the overall incidence was 27%, that is, higher than described in the literature (up to 12%) [2]. Maybe vocal cord injuries did not only occur during tracheal intubation but also during surgery and during removal of the tracheal tube. 

Volatile anesthetics increase neuromuscular block of muscle relaxants; anesthesia induction with desflurane increased neuromuscular block compared with a total intravenous anesthesia [3]. Thus, sevoflurane as part of the anesthesia would increase neuromuscular block; moreover, sevoflurane would lengthen neuromuscular block; vocal cords, therefore, would be longer relaxed. We speculated that sevoflurane would cause less vocal cord injuries than propofol during surgery and after removal of the tracheal tube.

After surgery, we assessed hoarseness, sore throat, and vocal cord injuries—by stroboscopy—up to 72 hours. We expected that the patients receiving sevoflurane would have had a lower incidence and severity of hoarseness and vocal cord injuries than the patients receiving an intravenous anesthesia with propofol.

## 2. Methods

The study was performed at the University Hospital of Rostock, Germany, between August 2010 and October 2011. Ethical approval for this study (registration number: A 2010 29) was provided by the Institutional Review Committee (Ethik-kommission der Universitat Rostock, Rostock, Germany) on 10 May 2010. The study was registrated at ClinicalTrialsGov under number NCT01616966. 

After obtaining written informed consent, we studied 65 adult patients, aged 18–80 yr, ASA I–III, undergoing orotracheal intubation for surgery of the ear. All patients were examined by stroboscopy one day before surgery and were excluded from the study when preexisting pathologies of the vocal cords were found. Exclusion criteria were obesity (defined as body mass index (BMI) > 40 kg/m²), an allergy against the study drugs, and patients with a known or suspected difficult airway (Mallampati score 3 or 4 and a mouth opening < 3.5 cm). 

### 2.1. Randomization and Monitoring

Patients were randomized—by a study nurse—in two groups, according to the randomization list, which was prepared via a computer-generated randomization program, as follows [4]: SEVO group, receiving sevoflurane during anesthesia, or TIVA group, receiving an intravenous anesthesia with propofol. We used neuromuscular monitoring to have comparable muscular block at time of intubation in both groups; neuromuscular monitoring was performed with the TOF Watch SX device (Organon Teknika, Eppelheim, Germany). We used neuromonitoring to have comparable depth of anesthesia between the study groups; neuromonitoring was performed with the BIS Vista brain monitoring system (Aspect Medical Systems, Norwood, MA, USA). 

The acceleromyographic measurements were done according to the guidelines of Good clinical research practice in pharmacodynamic studies of neuromuscular blocking agents from 2007 [5]. We used a calibrated TOF watch SX. After recalibration of the acceleromyography, rocuronium 0.45 mg/kg was injected over 5 s. Time to a train-of-four (TOF) ratio of 1.0 (defined as the time from start of injection of rocuronium until the TOF ratio reached 1.0) was measured. 

 The bispectral index (BIS) monitoring was applied according to the manufacturer's guidelines; BIS Quatro sensor electrodes were placed on the patient's forehead. The BIS values were noted continuously every minute during surgery. The target BIS was between 40 and 50. In the TIVA group, propofol infusion was increased from 4.0 mg/kg/h by 1.0 mg/kg/h than the BIS value was above 50; propofol infusion was decreased by 1.0 mg/kg/h than the BIS value was under 40 (minimum dosage was 4.0 mg/kg/h). In the SEVO group, sevoflurane was increased by 0.1 vol.% than the BIS value was above 50; sevoflurane was decreased by 0.1 vol.% than the BIS value was under 40 (minimum concentration was 1.0 vol.%).

### 2.2. Induction and Maintenance of Anesthesia

 One hour before the beginning of surgery, the patients received midazolam 7.5 mg orally. Induction was standardized for both groups. Remifentanil 0.4 *μ*g/kg/min was applied continuously for 2 min; afterwards, propofol 2.0 mg/kg was administered. After calibration of the TOF Watch SX, rocuronium 0.45 mg/kg was given. If the neuromuscular block was incomplete, rocuronium 0.15 mg/kg was added. Tracheal intubation was performed, when maximum block was achieved. All tracheal intubations were performed by the same anesthesiologist to control interindividual differences.

Maintenance of anesthesia was standardized; TIVA group: propofol 4.0 mg/kg/h and remifentanil 0.25–0.35 *μ*g/kg/min; SEVO group: sevoflurane 1.0 vol.% in a 50% oxygen-air mixture and remifentanil 0.25–0.35 *μ*g/kg/min. Fifteen minutes before the expected end of surgery, all patients received piritramide 0.05–0.1 mg/kg i.v. The patient's tracheas were extubated, when the TOF ratio was 1.0 and patients opened their eyes or began to cough; afterwards, the patients were moved to the postanesthesia care unit (PACU).

### 2.3. Assessment of Hoarseness, Sore Throat, and Vocal Cord Injuries

Hoarseness was defined as an acoustic quality that was different than the previous voice quality of the patient [6]. Sore throat was defined as continuous throat pain [7]. In the PACU and 24 h, 48 h, and 72 h after surgery, an investigator blinded to the group assignment of the patients assessed the incidence and severity of hoarseness and sore throat (see Appendix). If hoarseness or sore throat persisted over 72 h, a daily follow-up examination was performed until complete restitution. Vocal cord injuries were assessed by laryngostroboscopy by an ear-nose-throat physician who was unaware of the patient's group assignment (see Appendix); all examinations were performed by the same ear-nose-throat physician.

Compared to indirect laryngoscopy, laryngostroboscopy can detect functional disorders of the vocal cords, such as dysfunction of the mucosal wave. All patients were examined by stroboscopy 24 h after surgery. When hoarseness was lasting longer than 48 h, a second stroboscopy was performed 72 h after surgery. 

### 2.4. Assessment of Intubating Conditions, Intubating Variables, and Extubating Conditions

 The intubating conditions were assessed according to the consensus conference on Good Clinical Research Practice (GCRP) in Pharmacodynamic Studies of Neuromuscular Blocking Agents [5]. In addition, the following variables were assessed: Cormack's grades, time for intubation, the number of intubation attempts (see Appendix), and the extubating conditions—excellent = no coughing, good = slight coughing, and poor = sustained coughing during removing of the tracheal tube. The following factors were standardized: tube size (men: ID = 8.0 mm; women: ID = 7.0 mm), type of tube, and cuff inflation with air (cuff pressure was ≤25 mmHg) [1, 8].

### 2.5. Statistical Analysis

Statistical analysis was performed using the SigmaStat for Windows Version 3.5, (Systat Sotware Inc., San Jose, California, USA). Demographic data were analyzed using Mann-Whitney *U*-test or *t*-test; results are presented as mean (SD) or as median (range). Comparisons between groups were performed using the *χ*² test, Fisher's exact test, or Kruskal-Wallis' ANOVA test. Results were considered statistically significant, when *P* < 0.05. 

The sample size calculation was based on the study by Maruyama et al. [9]. The required number of patients for our study groups was calculated on the assumption that 55% of the patients suffered from hoarseness after a total intravenous anesthesia [9] and 16% after an anesthesia with desflurane [1]. For an 80% power and an *α* = 0.05, 52 patients (26 patients in each group) were needed. To compensate for possible drop-outs, we enrolled 59 patients (10% more than needed). 

## 3. Results

**Figure 1:**
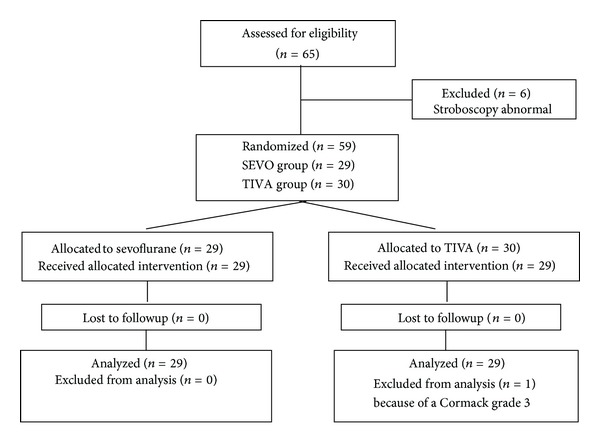
Flow diagram of patient distribution. Sevoflurane = anesthesia with sevoflurane and remifentanil. TIVA = anesthesia with propofol and remifentanil.

Six patients (10%) had preexisting pathologies at the vocal folds; consequently, these patients were excluded from the study. 59 patients were randomized for the study, 30 patients in the TIVA group, and 29 patients in the SEVO group. One patient—from the TIVA group—was excluded because of a Cormack grade 3 (Figure 1). There were no significant differences in the patient's characteristics (Table 1). Dosages of propofol, remifentanil, and rocuronium and end-tidal concentrations of sevoflurane are shown in Table 2.

### 3.1. Intubating Conditions, Intubating Variables, and Extubating Conditions

**Figure 2:**
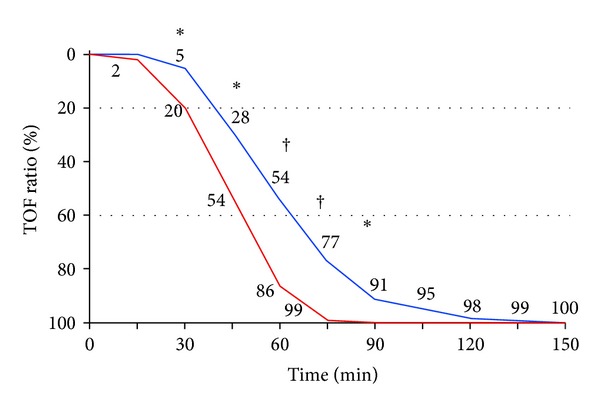
Mean TOF ratio values during surgery in patients receiving sevoflurane (SEVO group; upper blue line) or propofol (TIVA group; lower red line). *P = 0.003 SEVO group versus TIVA group. ^†^ *P* < 0.001 SEVO group versus TIVA group.

The patient's tracheas were extubated at a TOF ratio of 1.0. Patients in the SEVO group had a significantly longer neuromuscular block compared with the TIVA group (Table 3; Figure 2). Tracheal intubation was successful in all patients of both groups. Intubating variables and intubating conditions were comparable (not significant). Overall 39 patients (67%) had coughing during removal of tracheal tubes; 17 (59%) patients in the SEVO group versus 22 (76%) patients in the TIVA group; *P* = 0.26 (Table 3).

### 3.2. Hoarseness, Sore Throat and Vocal Cord Injuries

**Figure 3:**
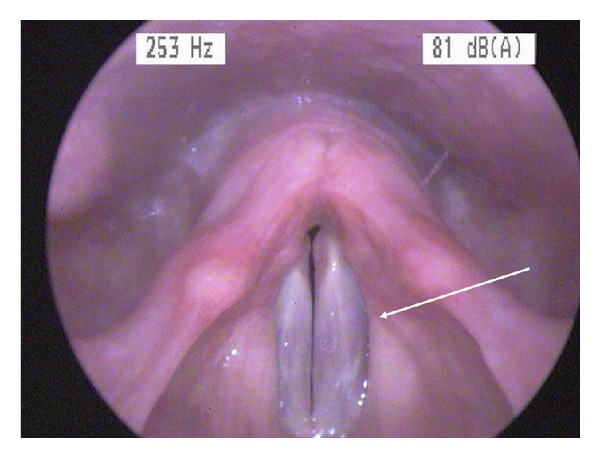
Edema of the left vocal cord (arrow) at 24 h after surgery (sevoflurane group).

The overall incidence (TIVA and SEVO groups together) of hoarseness was 10% (6 patients); the overall incidence of sore throat was 15% (9 patients). The incidence was comparable between groups (Table 4); the severity was comparable, too (data not shown). The overall incidence of vocal cord injuries after surgery was 24% (14 patients). There was no significant difference between the TIVA and SEVO groups: 5 versus 9 patients (*P* = 0.36). No patient suffered from hoarseness or had vocal cord injuries longer than 3 days. Vocal cord injuries were edema in 7 patients and erythema in 7 patients (Figure 3); there were no hematoma and no granuloma. The majority of vocal cord injuries were unilateral (left: 3; right: 6 patients); 5 patients had bilateral vocal cord injuries (1 patient; SEVO group versus 4; TIVA group; *P* = 0.35). A dysfunction of the mucosal wave was found in 20 patients: 8 patients in the SEVO group and 12 patients in the TIVA group. 

**Figure 4:**
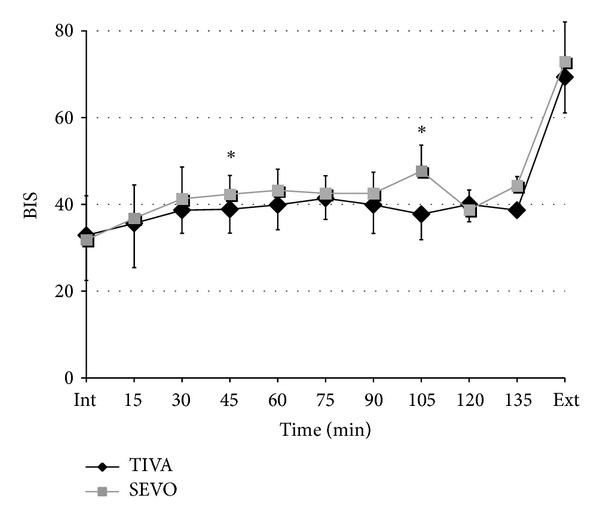
BIS values during surgery in patients receiving sevoflurane (gray square) or propofol (black diamond) (mean and SD). *P < 0.05 SEVO group versus TIVA group. BIS = bispectral index.

Postoperative nausea and vomiting (PONV) were not observed in the PACU. The BIS values are shown in Figure 4.

## 4. Discussion

We showed that both anesthesia techniques—anesthesia with sevoflurane and intravenous anesthesia with propofol—may be used. The rate of vocal cord injuries was comparable between the two anesthetics. The risk for postoperative residual curarization, however, was higher in the sevoflurane group. 

The incidence of hoarseness and sore throat was 10% in the TIVA group; this is lower than reported by Maruyama et al. [9]. He found hoarseness in 55% and sore throat in 50% of patients after a total intravenous anesthesia. The reason is, in our opinion, that the general anesthesia was maintained with propofol, fentanyl, and ketamine in his study. In our study, general anesthesia was maintained with propofol and remifentanil—a potent ultra short-acting synthetic opioid drug—which was applied continuously. Therefore, the depth of anesthesia, measured by BIS monitoring, was stable during surgery (between 29 and 42). BIS values between 45 and 60 are recommended for a balanced anesthesia [10]. An anesthesia with fentanyl as a bolus technique provides unstable consciousness states during surgery compared with remifentanil. 

The trauma causing laryngeal injury can occur on several occasions [11]: during tracheal intubation, during surgery, and during tracheal extubation. We standardized the anesthesia induction and tracheal intubation to control the risk factors for laryngeal injury. 

During surgery, the head was moved only slightly by the surgeon. The incidence of vocal cord injuries was 24% in the present study. Laryngeal injuries following tracheal intubation vary between 4% and 12% [12–14]; without muscle relaxant it was 42% [1]; with double-lumen tube it was 44% [15]; with intravenous anesthesia it was 27% [2]. We found only minor injuries such as erythema and edema at the vocal folds. The majority of the vocal cord injuries were unilateral with 65%, indicating a slight injury during tracheal intubation or resulting from the movement of the head during surgery of the ear. Bilateral vocal cord injuries—as a typical sign for an injury during removal of the tracheal tube—were observed only in 35%. It is difficult to determine, when the vocal cord injuries occurred. We performed tracheal intubation, when maximum neuromuscular block was achieved; therefore, we hope that the baseline injuries—caused by tracheal intubation—are comparable in both groups. Bilateral injuries occur when the vocal cords beat against the tracheal tube during surgery or during removal of the tracheal tube. The incidence of coughing at tracheal extubation was comparable between groups (59% SEVO group versus 76% TIVA group; *P* = 0.26); the incidence of bilateral injuries was comparable, too (1 patient SEVO group versus 4 TIVA group; *P* = 0.35). 

Inhalational agents may increase the neuromuscular block by a central mechanism; this may explain why the time of muscle relaxation in the SEVO group was increased compared with the TIVA group. Time to a TOF ratio of 1.0 was significantly longer in the SEVO group; doses of rocuronium were comparable between groups. Five patients in the TIVA group needed neostigmine to achieve a TOF ratio of 1.0; eleven patients in the SEVO group, however, needed neostigmine (*P* = 0.14); this is not significantly related, but this is clinically relevant. The risk for residual curarization is increased with sevoflurane. Residual curarization in the postanesthesia care unit is associated with an increased incidence of critical respiratory events, such as hypoxemia [16]. In our study, all patients were monitored with a calibrated TOF Watch SX; therefore, patient's tracheas were extubated, when the TOF ratio had reached 1.0. 

We demonstrated that sevoflurane did not decrease laryngeal morbidity; the risk for residual curarization, however, was higher compared with total intravenous anesthesia. Neuromuscular monitoring is—especially under sevoflurane anesthesia—necessary to detect residual neuromuscular blockade.

## Figures and Tables

**Table 1:** Demographic data, duration of surgery, and anesthesia.

	SEVO group (*n* = 29)	TIVA group (*n* = 29)	*P* value
Age (yr)	47 (17)	49 (15)	0.76
Weight (kg)	75.0 (14.2)	81.2 (16.8)	0.13
Height (cm)	172.2 (8.9)	173.5 (9.8)	0.61
Body mass index (kg/m²)	25.2 (4.1)	26.7 (3.1)	0.13
Gender ratio (female/male)	15/14	11/18	0.43
Smoking	10 (34%)	12 (41%)	0.78
Reflux	3 (10%)	4 (14%)	1.00
Duration of surgery (min)	75 (37)	73 (33)	0.87
Duration of anesthesia (min)	102 (36)	94 (34)	0.33

Values are mean (SD) or numbers (%). SEVO group: anesthesia with sevoflurane and remifentanil. TIVA group: anesthesia with propofol and remifentanil.

**Table 2:** Doses of rocuronium, propofol, remifentanil, and end-tidal concentrations of sevoflurane and administration of neostigmine.

	SEVO group (*n* = 29)	TIVA group (*n* = 29)	*P* value
Propofol for induction of anesthesia (mg)	150 (100–280)	170 (90–260)	0.37
Remifentanil (*µ*g/kg/min)*	0.256 (0.015)	0.259 (0.026)	0.98
Propofol (mg/kg/h)*	—	4.9 (0.8)	
Sevoflurane (vol.%)^†^	1.3 (0.3)	—	
Rocuronium (mg)	30 (25–55)	40 (25–70)	0.17
Administration of neostigmine (*n*)	11	5	0.14

Values are mean (SD), median (range), or numbers. SEVO group: anesthesia with sevoflurane and remifentanil. TIVA group: anesthesia with propofol and remifentanil. *Mean dosage during maintenance of anesthesia. ^†^Mean end-tidal concentration of sevoflurane during anesthesia.

**Table 3:** Neuromuscular measurements, intubating variables, and coughing during removal of the tracheal tube.

	SEVO group (*n* = 29)	TIVA group (*n* = 29)	*P* value
Neuromuscular measurements			
Time till TOF ratio = 1.0 (min)	71 (38–148)	52 (21–74)	<0.001
Time without relaxation (min)	14 (2–151)	34 (0–118)	0.03
Intubating variables			
Cormack grades 1/2	17/12	19/10	0.78
Time for intubation (s)	16 (9–170)	16 (9–26)	0.49
Attempts (*n*) 1/2/3	25/3/1	29/0/0	0.12
Extubating conditions			
Coughing	17 (59%)	22 (76%)	0.26

Values are median (range) or numbers (%). Time till TOF ratio = 1.0 (min) = time from start of injection of rocuronium until the TOF ratio reached 1.0. Time without relaxation = time from TOF ratio of 1.0 till tracheal extubation. SEVO group = anesthesia with sevoflurane and remifentanil. TIVA group = anesthesia with propofol and remifentanil.

**Table 4:** Incidence of hoarseness, sore throat, and vocal cord injuries.

	Hoarseness			Sore throat			Vocal cord injuries		
	SEVO (*n* = 29)	TIVA (*n* = 29)	*P* value	SEVO (*n* = 29)	TIVA (*n* = 29)	*P* value	SEVO (*n* = 29)	TIVA (*n* = 29)	*P* value
PACU	2	2	1.00	2	1	1.00	—	—	—
At 24 h	1	1	1.00	5	2	0.42	9	5	0.36
At 48 h	0	0	—	2	1	1.00	—	—	—
At 72 h	0	0	—	1	1	1.00	0	0	1.00
>72 h	0	0	—	0	1	1.00	—	—	—
Patients*	3 (10%)	3 (10%)	1.00	6 (21%)	3 (10%)	0.47	9 (31%)	5 (17%)	0.36

Values are shown as numbers of patients (%).*Patients = number of patients with hoarseness, sore throat, or vocal cord injuries (without dysfunction of mucosal wave). PACU: postanesthesia care unit. SEVO group: anesthesia with sevoflurane and remifentanil. TIVA group: anesthesia with propofol and remifentanil.

## Appendix

### Assessment of Hoarseness, Sore Throat, and Vocal Cord Injuries

(A) *Hoarseness* [1]. Do you have any hoarseness at all since your operation?

If the answer was no, hoarseness was graded 0 = none; if the answer was yes, hoarseness was recorded as follows: 1 = noticed by patient, 2 = obvious to observer, and 3 = aphonia.

(B) *Sore Throat* [7, 17]. Do you have any sore throat?

If the answer was no, sore throat was graded 0 = no sore throat; if the answer was yes, sore throat was recorded as follows: 1 = mild (pain with deglutition), 2 = moderate (pain present constantly and increasing with deglutition), and 3 = severe (pain interfering with eating and requiring analgesic medication).

(C) *Vocal Cord Injuries* [1, 2, 11]. Location: unilateral (left or right vocal cord) or bilateral (both vocal cords).

Type of injury: erythema = redness of the mucosa with surrounding inflammatory swelling, edema = swollen mucosa at the vocal folds, hematoma = bleeding into vocal cord, granuloma = granulation tissue remains as chronic, localized, one rounded tissue, and dysfunction of the mucosal wave = abnormal movement of the vocal cords.

(D) *Intubating Variables*. These variables are as follows.

- Time for intubation = time in seconds from the initial inserting of the laryngoscope into the patient's mouth until blocking of the cuff.
- Number of intubation attempts.
